# The surgical approach strategies in the treatment of anterior mediastinal tumors

**DOI:** 10.1080/07853890.2025.2609354

**Published:** 2026-03-31

**Authors:** Junwei Xie, Hongliang Wang, Tianci Han, Wei Tong, Xiaoqi Guo, Min Zhang, Dongzhe Liu, Hongxu Liu, Liang Zhang

**Affiliations:** ^a^ Department of Thoracic Surgery, Liaoning Cancer Hospital & Institute, Shenyang, China; ^b^ Department of Thoracic Surgery, Cancer Hospital of China Medical University, Shenyang, China; ^c^ Department of Thoracic Surgery, Cancer Hospital of Dalian University of Technology, Shenyang, China; ^d^ Department of Cardiothoracic Surgery, Tieling City Central Hospital, Tieling, China; ^e^ Department of Thoracic Surgery, Liaoning University of Traditional Chinese Medicine, Shenyang, China; ^f^ Department of Thoracic Surgery, Central Hospital Affiliated to Shenyang Medical College, Shenyang, China; ^g^ Department of Cardiothoracic Surgery, Kuandian Manzu Autonomous County Central Hospital, Dandong, China

**Keywords:** Anterior mediastinal tumors, surgical approaches, treatment outcomes, thymomas, clinical condition, technology

## Abstract

**Background:**

Anterior mediastinal tumors, involving structures such as the thymus and lymph nodes, pose significant clinical challenges due to their asymptomatic nature and potential to cause severe symptoms as they grow or invade surrounding organs. Treatment varies by tumor type, with surgery being crucial, especially for benign tumors and thymomas. The complex anatomy of the anterior mediastinum makes surgical approach selection critical for treatment outcomes.

**Method:**

A comprehensive literature search was conducted to identify studies related to surgical approaches for anterior mediastinal tumors. Two major databases, PubMed and Web of Science, were searched for studies published between 2000 and 2025. Key search terms included ‘anterior mediastinal tumors’, ‘thymoma’, ‘Robot-Assisted Surgery’, ‘Video-Assisted Thoracoscopic Surgery (VATS)’, and ‘surgical approach’. Inclusion criteria were limited to clinical studies, reviews, and case series reporting on the surgical management of anterior mediastinal tumors in adults. Studies focusing on non-surgical treatment or lacking sufficient clinical outcome data were excluded. Due to wide variations in study designs, patient populations, and surgical techniques, a formal meta-analysis was not performed. Instead, a narrative review of the available literature was conducted to provide a comprehensive qualitative summary of current surgical strategies, their indications, and outcomes. This study explores various surgical approaches for anterior mediastinal tumors, including traditional open methods (e.g. Median Sternotomy) and minimally invasive techniques (e.g. Thoracoscopic and Robot-Assisted Surgery). It analyzes their indications, operative difficulty, complication rates, and postoperative recovery, integrating the latest technological advancements.

**Results:**

Different surgical approaches have distinct characteristics. Traditional open surgeries offer clear exposure but result in significant trauma and complications. Minimally invasive techniques, such as Thoracoscopic Surgery, provide advantages like smaller incisions, reduced recovery time, and fewer complications, but require advanced technical skills and may have limited applicability for certain tumor locations and sizes. Robot-Assisted Surgery combines minimally invasive benefits with high precision and better visualization, though its high cost limits widespread use.

**Conclusion:**

The choice of a surgical approach should be personalized based on the tumor’s location and size, the patient’s clinical condition, and expected postoperative outcomes. For small, accessible tumors, Thoracoscopic Surgery is preferred; for complex or larger tumors, open or Robot-Assisted Surgery may be more suitable. Future advancements in technology and multidisciplinary collaboration are expected to further improve treatment outcomes for anterior mediastinal tumors.

## 1. Introduction

Anterior mediastinal tumors are a common type of mediastinal tumor, located in the midline of the chest cavity and primarily involving the thymus, lymph nodes, blood vessels, and other soft tissue structures. The most common tumor types in the anterior mediastinum are thymoma, lymphoma, and cysts. Surgery is a key treatment for anterior mediastinal tumors [1]. Due to the complex anatomy of this region, selecting an appropriate approach is vital for achieving effective outcomes. Surgeons must carefully avoid injury to major vessels, nerves, and cardiopulmonary structures, which makes the choice of surgical pathway a major challenge.

In recent years, minimally invasive techniques, such as VATS and robotic surgery, have become increasingly important in the management of anterior mediastinal tumors. These techniques offer potential advantages, including reduced recovery times and lower complication rates, making them highly relevant in current surgical practice. This article aims to explore surgical approaches for anterior mediastinal tumors and evaluate their respective benefits and limitations.

## 2. Anatomical features of the anterior mediastinum

### 2.1. Anatomy of the mediastinum

The mediastinum is located centrally within the thoracic cavity, separating the space between the left and right lungs and lying adjacent to the chest wall, spine, and diaphragm. It is divided into three parts: the anterior, middle, and posterior mediastinum. The anterior mediastinum is situated posterior to the sternum, in the anterior part of the thoracic cavity, and primarily contains the thymus [2], adipose tissue, certain major blood vessels, and lymph nodes. Its upper boundary is the lower edge of the sternum, and its lower boundary is the diaphragm. The anatomical structure of the anterior mediastinum is complex, involving numerous vital blood vessels, nerves, and organs. Any damage to these structures can result in serious clinical consequences.

### 2.2. Clinical presentation of anterior mediastinal tumors

The clinical manifestations of anterior mediastinal tumors depend on tumor size, location, and compression of adjacent structures. Small tumors may be asymptomatic and are often detected incidentally on imaging studies, while larger tumors commonly cause respiratory symptoms (e.g. cough, dyspnea, airway obstruction) when the trachea or bronchi are involved [3]. Compression of major vessels, especially the superior vena cava, can lead to superior vena cava syndrome, which presents with facial and upper limb edema, and in severe cases, hypotension or syncope. Cardiac compression may trigger chest pain or palpitations, whereas esophageal involvement can result in dysphagia, odynophagia, or retrosternal discomfort during eating, occasionally leading to aspiration. Neural involvement is also common: compression of the vagus nerve or sympathetic chain may cause hoarseness, cough, altered breath sounds, or facial flushing [4].

## 3. Surgical approaches for anterior mediastinal tumors

### 3.1. Median sternotomy approach

#### 3.1.1. Traditional median sternotomy approach

**Figure 1.**
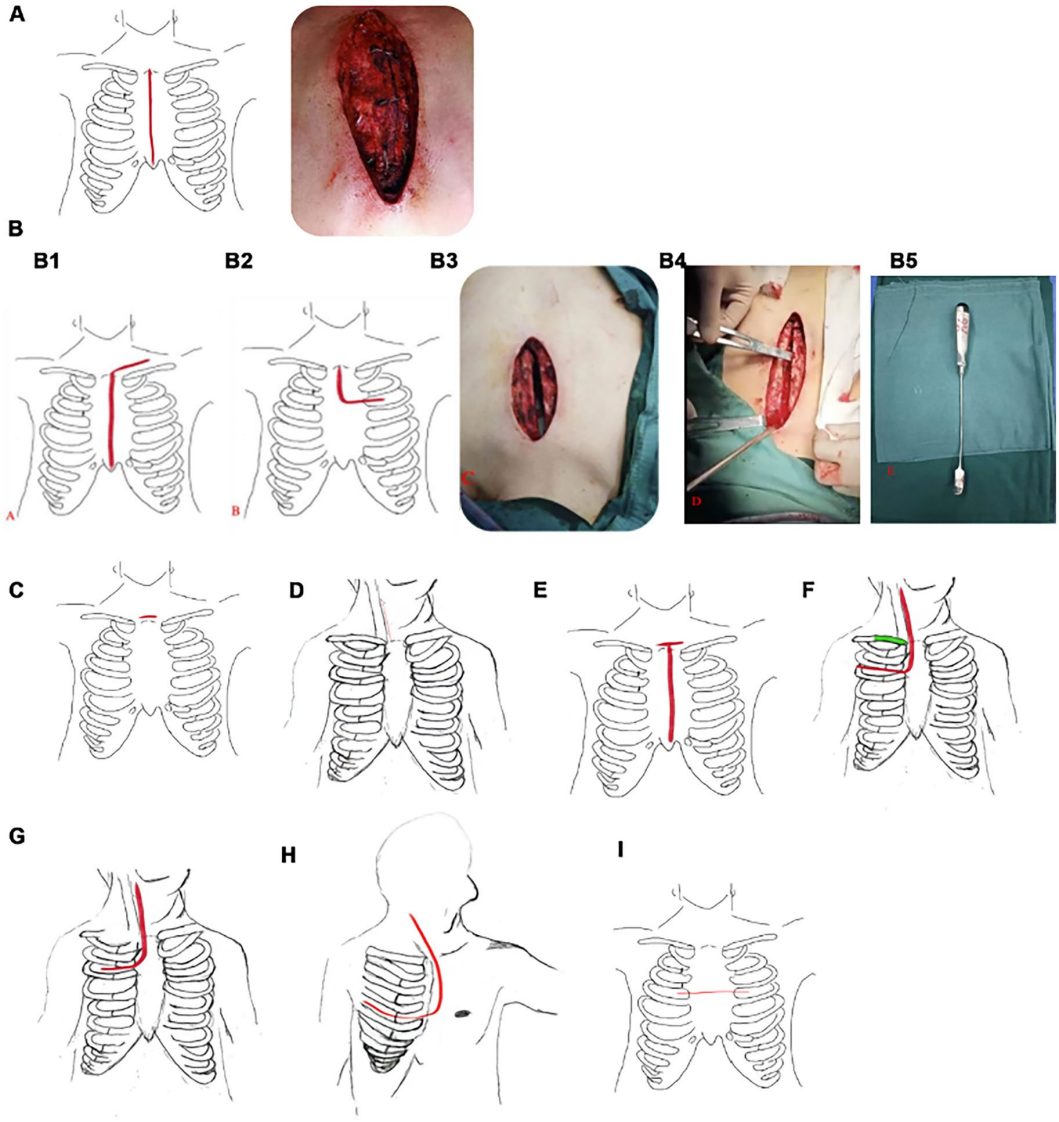
The surgical approach. (A) The figure shows a median incision approach, splitting along the median sternum. (B) Longitudinal incision in the center of the sternum from top to bottom. B1 shows a reverse L-shaped incision, B2 shows an L-shaped incision, B3 shows the L-shaped incision during surgery, B4 shows ⊥ incision, B5 shows the ion stripping behind the sternumkocher incision. (C) Kocher incision. (D) Small longitudinal incision in the neck. (E) T-shaped incision on the neck. (F) The red line is the Dartevelle incision, and the green part is the excised clavicle. (G) Grunenwald incision. (H) Hemi-Clam Shell incision. (I) Transverse sternum between ribs 3. All schematic diagrams of surgical techniques were hand-drawn by the authors, and the surgical photographs were captured by the authors during clinical procedures.

Median sternotomy is the standard surgical approach for anterior mediastinal tumors (Figure 1(A)). It provides clear exposure, a large operative field, and minimal instrument interference, making it suitable for most tumor types. For large superior mediastinal tumors, this approach facilitates protection of critical structures (e.g. the brachiocephalic veins, superior vena cava, and phrenic nerves) and allows for major vascular reconstruction when required.

Despite these advantages, the approach has notable drawbacks: significant trauma, blood loss, postoperative pain, and impaired cardiopulmonary function, which increase the risk of complications and mortality, particularly in elderly or myasthenic patients [5]. It also disrupts thoracic cage stability, raising the risk of the sternum, mediastinum, and pulmonary infections. Comparative studies have reported a complication rate in sternotomy cases, higher than other approaches [6]. Grossi’s analysis of 7,949 patients revealed 77 cases (0.97%) of sternal wound infections, which markedly delayed recovery [7].

The surgical procedure involves dividing the sternum with wires or an electric saw, opening it with a sternal retractor, and excising the tumor and surrounding fat under direct vision. The sternum is reapproximated and stabilized with steel wires. When minimally invasive surgery is not feasible, median sternotomy remains the most reliable option [8].

#### 3.1.2. Upper sternum midline L-shaped, reverse L-shaped, or ‘⊥’ half Sternotomy approach

The upper sternum midline L-shaped (Figure 1(B2 and B3)), reverse L-shaped (Figure 1(B1)), or ‘⊥’-shaped (Figure 1(B4)) half sternotomy combines longitudinal and transverse incisions to achieve broader exposure of the anterior mediastinum. Typically, the sternum is divided vertically from the sternal notch to the third or fourth rib, then horizontally, with partial opening of the intercostal space. A sternal retractor is used to provide full tumor exposure.

**Figure 2.**
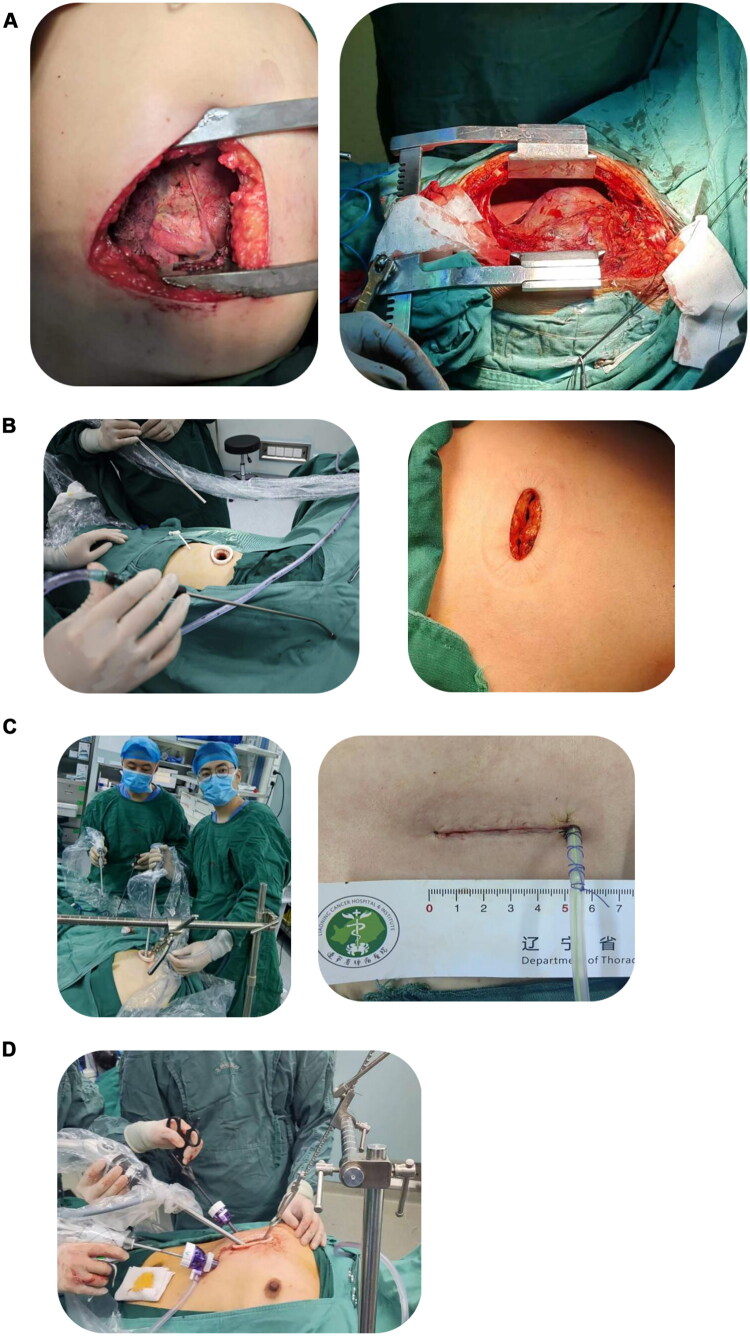
The surgical approach. (A) As can be seen from the figure, one side thoracotomy for mediastinal mass resection has a large incision, but the surgical field is clear and the operation is convenient and safe. (B) The endoscopic device entering the chest through the right side, the incision on the left is about 5 cm in size. (C) The left figure shows the subxiphoid ‘single hole’ method, and the right figure shows the incision size after surgery. (D) ‘Three-hole’ method under xiphoid process. It can be seen from the figure that the sternal retractor is needed to pull up the sternum to increase the operation space. All surgical photographs were captured by the authors during clinical procedures.

The L-shaped incision extends laterally, facilitating dissection of pulmonary vessels at the hilum and enabling pulmonary tumor resection with lymph node dissection. This approach avoids clavicle resection, preserves the sternoclavicular joint, and maintains shoulder stability and cosmetic appearance, thereby reducing postoperative discomfort [9]. It also offers clearer exposure of major vessels at the cervicothoracic junction, particularly the distal subclavian vessels, and facilitates mediastinal lymph node clearance. However, posterior thoracic inlet structures remain inadequately exposed [10].

A ‘⊥’ incision (Figure 1(B4)), though less common, combines the advantages of the L-shaped and reverse L-shaped approaches, providing wider exposure and greater safety. In practice, a sternal elevator (Figure 1(B5)) may be used to separate the retrosternal space, but both sternal sides must be fixed to prevent instability. If intraoperative bleeding or adhesions hinder exposure, the incision can be extended downward to convert into a full Median Sternotomy, offering greater flexibility.

### 3.2. Neck incision approach

#### 3.2.1. Small neck incision approach

The small neck incision approach, usually the Kocher incision above the sternal notch (Figure 1(C)), is designed for tumors at the cervicothoracic junction. The patient is placed in a supine position with the shoulders elevated and neck extended. An arc-shaped incision is made along the skin crease above the sternal notch. The subcutaneous tissue is divided, and when necessary, the platysma, sternohyoid, and sternothyroid muscles are bluntly separated or divided to expose the retrosternal space. Adequate exposure of the anterior mediastinum is then achieved without entering the thoracic cavity, thus preserving sternal stability and minimizing pulmonary impact.

This approach is most commonly used for retrosternal thyroid tumors, which usually descend along the tracheal space while retaining blood supply from the inferior thyroid artery. The incision allows clear visualization and ligation of thyroid arteries, reducing the risk of recurrent laryngeal nerve injury. The thyroid is then mobilized by suture traction or digital blunt dissection from the retrosternal space and gently delivered into the mediastinum for complete removal. This technique is simple, minimally traumatic, and time-efficient. In cases with variant mediastinal vessels, partial sternal division may be necessary for safety. National Surgical Quality Improvement Program (NSQIP) data showed that, under proper conditions, the trans-cervical approach had fewer complications than trans-thoracic surgery, which is associated with higher mortality [11].

Beyond thyroid tumors, this incision can also be used for resecting high-positioned thymus tissue and anterior mediastinal lesions. Since Sauerbruch first performed cervical thymectomy for myasthenia gravis in 1912 [12], further studies have demonstrated the feasibility of complete thymectomy through this approach using standard instruments and a headlamp [13]. However, we believe this method should be reserved for early-stage, small tumors without myasthenia gravis and when extensive adipose clearance is unnecessary.

#### 3.2.2. Mediastinoscopy-assisted operation

After anesthesia, the patient’s shoulders and back are elevated. A transverse incision is made along the skin crease at the sternal notch. Through blunt dissection, the surgeon reaches the anterior trachea and further separates the trachea, brachiocephalic vessels, and posterior aortic arch. A posterior tunnel is then created, and a mediastinoscope is inserted to perform the operation. Compared with the small neck incision, mediastinoscopy provides a clearer and deeper operative field, exposing vascular pathways more distinctly. Vascular clamps or ultrasonic scalpels may be used, though instrument interference within the mediastinoscope and the smaller operative range compared with thoracoscopy increase the technical difficulty.

Despite these challenges, mediastinoscopy has unique advantages in vascular surgery. It enables deeper entry into the thoracic cavity with clear visualization of vessel anatomy, facilitating accurate evaluation and treatment of vascular lesions. Additionally, it is minimally invasive, associated with faster recovery, and reduces surgical risk. Studies have reported that mediastinoscopic surgery shortens operative time, decreases postoperative complications, reduces infection rates, and shortens hospital stays compared with conventional surgery [14–16].

#### 3.2.3. Longitudinal small neck incision approach

The longitudinal small neck incision approach (Figure 1(D)) is mainly used for tumors at the cervicothoracic junction, particularly when the mass is primarily located in the neck and its blood supply originates from cervical vessels. After making a vertical incision above the sternal notch, blunt dissection of the inferior tumor border allows safe mobilization and complete resection. This incision provides clear exposure of tumors in the cervicothoracic tumors, facilitates identification and protection of cervical vessels and nerves, reduces the risk of intraoperative bleeding, and lowers the incidence of complications. The smaller incision also results in less trauma, faster recovery, and shorter hospital stays.

A large retrospective study of 11,849 patients with thyroid goiters confirmed that most blood supply arises from cervical arteries, enabling treatment through a cervical incision in the majority of cases, with fewer complications and shorter hospitalization. The cervical approach has also been shown to be effective for multinodular goiters, with trans-thoracic or sternotomy procedures needed only when the cervical route is insufficient [17].

However, limitations exist. Tumor adhesion to surrounding tissues, especially in large or deep lesions, increases the risk of nerve and vessel injury. The technique requires advanced surgical skills for safe dissection and removal. Moreover, its resection range is limited: while suitable for superficial tumors, deeper or multiple tumors may require alternative or combined surgical approaches [18].

### 3.3. Neck-chest anterior incision approach

#### 3.3.1. Anterior ‘T’-shaped neck incision

When performing the anterior ‘T’-shaped neck incision (Figure 1(E)), an arcuate incision is first made at the anterior neck, followed by a vertical midline incision from its midpoint. The length of the vertical cut depends on tumor size, extending to the second rib cartilage or even to the xiphoid process; larger tumors require longer incisions. Partial sternal splitting allows full exposure of cervical vessels and nerves.

This approach is mainly used for cervicothoracic junction tumors that cannot be safely removed through a cervical incision alone, such as retrosternal thyroid tumors extending below the aortic arch. These tumors may compress major vessels, particularly the superior vena cava, or even the lung lobes, producing signs like the ‘iceberg sign’ or ‘pear shape’ [19].

The main advantage of the ‘T’-shaped incision is its excellent exposure, offering a broad and clear view of both cervical and thoracic structures. It is especially beneficial for large or complex tumors closely associated with vital vessels and nerves, improving surgical precision and enabling complete resection while reducing recurrence and complications. Studies have shown that this approach facilitates full removal of thyroid tumors below the aortic arch, avoiding postoperative risks [19,20].

However, disadvantages include greater trauma due to the extended incision and sternotomy, longer recovery, and higher risks of bleeding or infection. The operation is technically demanding, requiring precise handling of vessels and nerves, and the larger incision may leave a more visible scar. Despite these limitations, the anterior ‘T’-shaped incision remains valuable for large or malignant tumors requiring extensive exposure.

#### 3.3.2. Dartevelle incision

The Dartevelle incision, first described by Dartevelle et al. in 1993, is designed for tumors at the cervicothoracic junction, particularly those involving the thoracic inlet (Figure 1(F)) [21]. The incision starts along the anterior border of the sternocleidomastoid muscle and extends laterally along the second intercostal space, with resection of the medial clavicle to expose the thoracic apex.

Its main advantage is the wide exposure of key structures, including the subclavian vessels and brachial plexus, facilitating safe tumor dissection and enabling subclavian vascular reconstruction when needed. It can also provide access for partial vertebrectomy in cases of anterior vertebral invasion. This approach is especially useful for large or complex tumors requiring extensive resection [22].

Clinical studies confirm its effectiveness. In Mazel et al’.s (2003) study, 36 patients with cervicothoracic tumors underwent radical resection with vertebrectomy, demonstrating the approach’s feasibility [23]. Other reports showed satisfactory outcomes in the resection of cervicothoracic paravertebral tumors, with maximal tumor clearance and low complication rates [24].

However, disadvantages include the removal of part of the clavicle, pectoralis major, and sternocleidomastoid attachments, which can cause chest wall asymmetry, shoulder dysfunction, and prolonged recovery. Additional risks include postoperative pain, limited mobility, and potential nerve or vessel injury.

Overall, the Dartevelle incision provides excellent exposure for tumors at the cervicothoracic junction, especially when vascular reconstruction or vertebral resection is required. Due to its invasiveness, it should be reserved for patients who need precise dissection and can tolerate greater surgical trauma.

#### 3.3.3. Grunenwald incision (anterior ‘L’-shaped incision)

The Grunenwald incision, first described by Grunenwald et al. in 1997, is a specialized surgical approach for tumors involving the thoracic apex (Figure 1(G)) [25]. The incision begins along the anterior border of the sternocleidomastoid muscle, extends vertically through the sternoclavicular joint to the first and second intercostal spaces, and then courses laterally along the deltopectoral groove to the mid-clavicular region. The first and second costal cartilages are divided, and the clavicle is mobilized by releasing its attachments to the scalene muscles and ligaments. The clavicle is then elevated while preserving the sternoclavicular joint.

This approach provides excellent exposure of critical cervicothoracic structures, including the subclavian vessels, brachiocephalic vein, carotid arteries, vagus nerve, brachial plexus, and upper thoracic vertebrae. It is particularly useful for apical tumors encasing the brachial plexus or subclavian vessels, as well as malignant lesions involving the subclavian or brachiocephalic origins, such as superior sulcus tumors and thymic carcinoma. The incision also affords access for partial vertebrectomy in selected patients.

Several advantages have been reported. The incision allows wide and direct visualization of the thoracic inlet [23], ensuring safe and complete resection of complex tumors. Clinical series have demonstrated higher resection success rates and significantly lower postoperative complication rates compared with alternative approaches [26]. Preservation of the clavicle and sternoclavicular joint minimizes impairment of shoulder and upper extremity function while maintaining chest wall symmetry and cosmetic appearance.

Nonetheless, the Grunenwald incision has limitations. Recovery tends to be prolonged, and restoration of shoulder mobility may take time. In cases requiring hilar vessel dissection, mediastinal lymphadenectomy, or resection of chest wall tumors below the second rib, exposure may be inadequate, necessitating additional incisions. Moreover, the approach is technically demanding, particularly when operating at the thoracic inlet, and should be performed by surgeons with advanced expertise.

#### 3.3.4. Hemi-Clam shell incision

The Hemi-Clam Shell incision, first described by Korst et al. in 1998 [5,27], is primarily used for resection of complex apical chest tumors (Figure 1(H)). The patient is placed in a supine position with shoulders elevated and arms extended laterally. The procedure begins with a cervical incision at the base of the neck, dividing the sternocleidomastoid and omohyoid muscles to expose lateral cervical structures. The incision is then extended inferiorly along the midline sternum and laterally into the third or fourth intercostal space, with possible extension to the axilla depending on tumor location. Additional resection of the second to fourth ribs or partial claviculectomy may be required for adequate exposure, particularly when the subclavian artery or brachial plexus is involved.

This approach provides excellent exposure of the subclavian artery, superior vena cava, brachiocephalic vein, and other critical structures, making it especially valuable for invasive tumors requiring vascular control, ligation, or reconstruction. It is well suited for malignant tumors involving the chest wall, great vessels, and nerves, ensuring radical resection under direct visualization.

Advantages include its ability to expose deep-seated apical tumors and adjacent vascular structures, thereby improving surgical accuracy and enabling safe vascular reconstruction when necessary. Clinical studies support its efficacy: in Korst’s original series of 42 patients, complete resection was achieved in 95%, major complications occurred in 9.5%, and the 5-year survival rate was 67.4% [27]. Kurisu later reported its successful use for the repair of ductal aneurysms in elderly patients, highlighting its flexibility in complex thoracic aortic surgery [28].

However, disadvantages include significant surgical trauma due to the extensive incision and rib or clavicle resection. Postoperative pain, delayed recovery of chest wall and shoulder function, and higher rates of complications (e.g. bleeding, infection, thrombosis, and nerve injury) are notable concerns. Mortality is also increased in cases with vascular invasion, necessitating meticulous surgical technique.

In summary, the Hemi-Clam Shell incision provides unparalleled exposure for large, invasive apical tumors requiring vascular reconstruction, but its use should be carefully weighed against the greater morbidity and prolonged recovery associated with this approach [29,30].

### 3.4. Transverse sternotomy approach

The transverse sternotomy approach involves a horizontal division of the sternum at the second or third intercostal space, followed by sternal retraction to expose the anterior superior mediastinum (Figure 1(I)). This technique, though less frequently used, has unique applications, particularly in mediastinal tumor resection and complex aortic replacement surgeries [31–34].

Its main advantages include providing a wider surgical field and superior exposure of deep mediastinal structures, making it especially valuable for the resection of large or deeply located mediastinal tumors where traditional vertical sternotomy may be limited. In vascular surgery, it offers sufficient space for precise aortic reconstruction or replacement, thereby improving operative accuracy and success rates.

However, transverse sternotomy is associated with significant drawbacks. The horizontal division of the sternum causes greater surgical trauma, leading to severe postoperative pain, prolonged recovery, impaired chest wall function, and a higher risk of poor sternal healing. Complications such as bleeding, infection, nerve injury, and chest wall deformity are more common, especially in elderly or frail patients. Bridgewater’s comparative study showed that transverse sternotomy was associated with longer operative time, higher complication rates, and increased mortality compared with traditional vertical sternotomy [35].

In summary, while rarely used, the transverse sternotomy approach provides excellent exposure and operative space in selected cases, particularly for deep mediastinal tumors and complex aortic procedures, though its morbidity limits its widespread adoption.

### 3.5. Axillary incision approach

#### 3.5.1. Left or right thoracotomy approach

The left or right thoracotomy approach involves entering the chest through the third or fourth intercostal space (Figure 2(A)). This incision provides reliable exposure of mediastinal tumors and the brachiocephalic vein, helping to reduce the risk of vascular injury. In complex cases (e.g. vascular damage, massive bleeding, large tumors, or severe adhesions), a transverse sternotomy can be added to expand the operative field and improve safety. The choice of side depends on the tumor location and its relation to adjacent neurovascular structures. Both approaches allow effective thymic exposure and mediastinal tumor resection, but each has specific strengths.

The right thoracotomy avoids interference from the heart and aortic arch, offering a clearer view of the right mediastinum. It also facilitates dissection of the right phrenic nerve, superior vena cava, and thymic vessels, making it particularly suitable for right-sided thymic tumors. Furthermore, it is ergonomically advantageous for right-handed surgeons, improving comfort and efficiency with a relatively short learning curve. However, this approach is limited in exposing left-sided lesions and is less effective for resections requiring extensive access to the left mediastinum.

The left thoracotomy provides better visualization of the left phrenic nerve and improved access for the resection of left-sided thymic tumors, fat tissue, or upper-pole lesions of the left thymus. It also facilitates surgery involving the aorta and its branches. Its limitations include restricted space due to the heart and aortic arch, which complicates manipulation of thymic vessels and reduces visibility in cases requiring wide exposure.

Mao et al. [34] compared left and right thoracotomy for anterior mediastinal tumor resection and found that the right-sided approach resulted in less postoperative drainage and shorter hospital stays. The authors suggested that the heart’s leftward position complicates the left-sided approach by limiting the operative field.

In summary, thoracotomy provides excellent access for mediastinal and thymic tumors as well as aortic procedures. The right approach is favored for right-sided tumors and aortic surgery, while the left approach is better suited for left mediastinal lesions. Surgical selection should be individualized based on tumor location, technical difficulty, and patient condition.

#### 3.5.2. Thoracoscopic-assisted intercostal approach

The Thoracoscopic-Assisted Intercostal Approach involves a 3–4 cm incision between the ribs, with thoracoscopy providing assistance (Figure 2(B)). This technique can be performed unilaterally or bilaterally, aiming to achieve the same extent of resection as open thoracotomy while minimizing trauma. Thoracoscopy provides excellent visualization, reduces postoperative pain, and shortens recovery time.

It is particularly suitable for small anterior mediastinal tumors (e.g. thymomas, thymic cysts) and posterior mediastinal tumors (e.g. neurofibromas) without significant adhesions. In these cases, thoracoscopy offers sufficient exposure with less trauma, and studies of 129 patients with anterior mediastinal tumors showed significant reductions in operative time, blood loss, drainage duration, hospital stay, and analgesic use compared with open surgery [34,36].

The main limitations include difficulty identifying the contralateral phrenic nerve and limited ability to remove contralateral fat or tumor tissue, which may leave residual disease. Visualization is also restricted at the cervicothoracic junction, increasing the risk of vascular or nerve injury, especially in cases with dense adhesions. For larger or invasive tumors, practical intraoperative triggers for conversion include uncontrolled venous or arterial bleeding, inadequate cranial exposure of the mass or great vessels, and concern for impending capsular rupture. In such cases, conversion to Median Sternotomy or extended anterolateral thoracotomy is recommended.

In summary, the thoracoscopic-assisted intercostal approach is an effective, minimally invasive option for small, non-adherent mediastinal tumors, providing precise visualization and faster recovery, though its utility is limited in large or invasive tumors.

#### 3.5.3. Da vinci robot-assisted lateral intercostal approach

The da Vinci robotic surgical system has recently emerged as an innovative option for mediastinal tumor resection. Yoshino et al. first reported its use in 2002, followed by Bodner et al. in 2004, both confirming its feasibility and safety [37]. The Robot-Assisted Lateral Intercostal Approach is particularly suitable for tumors closely related to surrounding structures, offering precise dissection in anatomically complex and highly vascular regions, thereby reducing complications.

Key advantages include high-definition three-dimensional visualization and robotic arms with a greater range of motion than the human wrist, enabling refined and flexible maneuvers. Augustin [38] reported 33 cases, with a 91% completion rate, mean operative time of 2 h, and no surgery-related mortality or major complications. Similarly, Aoshima’s series of 141 cases confirmed the safety and feasibility of robotic procedures [39], with resection results comparable to open surgery [40].

Limitations include the need for multiple incisions, longer operative times compared to single-port thoracoscopy, and lack of tactile feedback, which may increase the risk of vascular injury. Furthermore, the high cost of equipment and maintenance limits widespread adoption. Despite these drawbacks, the da Vinci system offers unique benefits in mediastinal tumor resection, and with advancing technology and decreasing costs, its clinical role is expected to expand. In robotic cases, similar bailout criteria apply: persistent bleeding that cannot be safely controlled, loss of a safe working space, or inadequate visualization of the innominate vein, superior vena cava (SVC), or phrenic nerve should prompt early conversion to Median Sternotomy or, when the thoracic inlet is involved, to an appropriate cervicothoracic incision.

### 3.6. High posterolateral incision approach (Shaw-Paulson incision)

The high posterolateral approach, also known as the Shaw-Paulson incision, is a long-established technique widely employed for the resection of posterior superior mediastinal tumors [41]. It provides excellent exposure of the posterior thoracic wall, mediastinum, thoracic spine, nerve roots, and pulmonary hilum, making it particularly valuable for complex resections. Its limitation lies in the inadequate visualization of the subclavian vessels, brachial plexus, and certain upper thoracic structures [27].

For this approach, the patient is positioned in the lateral decubitus position with the affected side up and the ipsilateral arm extended overhead and secured. The incision begins at the spinous process, extends along the medial border of the scapula to the level of the seventh cervical vertebra, then proceeds through the trapezius muscle to the first or second ribs. It continues downward, curving around the inferior angle of the scapula, and terminates at the anterior axillary line. Rib resection may be required to access the thoracic cavity. Once the cavity is entered, the tumor is dissected free from chest wall adhesions, adjacent pulmonary vessels are exposed, and involved lymph nodes are resected. This provides broad exposure of the posterior thoracic wall, transverse processes, thoracic nerve roots, and hilum, enabling complete tumor excision and lymphadenectomy.

The Shaw-Paulson incision is indicated for posterior superior mediastinal tumors invading the chest wall, spine, nerve roots, or pulmonary hilum, particularly when dense adhesions or vertebral involvement are present. It is advantageous for large tumors closely related to the thoracic spine or neurovascular structures, as the wide surgical field reduces the likelihood of residual disease and lowers the risk of local recurrence.

However, the approach has notable drawbacks. It provides limited exposure of the subclavian vessels, increasing the risk of catastrophic hemorrhage if these vessels are invaded. Exposure of the upper chest wall may also be inadequate in cases of bulky tumors or severe adhesions, complicating the determination of resection margins. In addition, the restricted access makes brachial plexus release challenging, which poses risks when nerve preservation is required.

### 3.7. Thoracoscopic sub-xiphoid approach

The thoracoscopic sub-xiphoid approach offers distinct advantages, particularly in delineating the relationship between anterior mediastinal tumors and major vessels such as the superior vena cava, brachiocephalic vein, brachiocephalic trunk, and intrathoracic arteries and veins. It also allows complete removal of anterior pericardial fat with clear identification of both phrenic nerves and their accompanying vessels, thereby minimizing the risk of injury to vital structures.

The patient is placed in a supine position, and a small incision is made below the xiphoid process and along the costal margin. Using thoracoscopy, the surgeon gradually enters the anterior mediastinum, carefully dissecting the tumor while assessing its relationship with adjacent vessels. A sternal retractor is often used to elevate the xiphoid and expand the retrosternal space, increasing operative exposure. This approach is especially suitable for the resection of anterior mediastinal tumors, most commonly thymomas, and is advantageous when pericardial fat dissection is required.

Compared with Median Sternotomy, the Subxiphoid Approach is minimally invasive, avoiding injury to intercostal muscles, nerves, and vessels, thus significantly reducing postoperative pain and facilitating recovery. Since Suda et al. [5] first reported single-port subxiphoid thymectomy in 2012 (Figure 2(C)), multi-port modifications (Figure 2(D)) have been widely adopted due to their easier technical feasibility.

Clinical studies confirm its benefits. A 2020 retrospective study of 238 patients, including 40 treated *via* the Subxiphoid Approach, demonstrated lower pain scores (VAS at 12–24 h: 4.36 vs. 2.23, *p* = 0.03) and shorter hospitalization durations compared with the intercostal approach [34]. Robot-Assisted Subxiphoid Thymectomy has also shown shorter operative times, reduced drainage duration, and less pain compared with lateral thoracic approaches [42]. However, other studies have found no significant differences in postoperative pain or stress compared with traditional approaches [43].

Limitations include the need for intraoperative cardiac compression, which increases risk in elderly patients and those with impaired cardiac function. Body habitus—such as obesity, extreme thinness, or pectus excavatum—may restrict operative space, making the procedure technically difficult. When tumors invade major vessels or are densely adherent to the pericardium, trachea, or surrounding tissues, surgical risk increases, and early conversion to Median Sternotomy or an intercostal VATS/open thoracotomy should be undertaken to ensure safe vascular control and avoid capsular rupture [36].

### 3.8. Combined approach

The combined surgical approach integrates multiple access routes to overcome the limitations of a single incision, particularly when the retrosternal space is narrow or the tumor spans multiple anatomical regions (e.g. neck, mediastinum, upper chest). Its key advantage is the expanded operative field, which enhances flexibility, facilitates safer dissection of tumors adjacent to vital structures such as the superior vena cava, brachiocephalic vein, and brachiocephalic trunk, and improves the likelihood of complete resection. For large or multi-regional tumors, this approach provides superior visibility and greater resection safety.

By combining multiple incisions, surgeons can achieve more efficient tumor exposure and dissection, thereby reducing operative time. One study reported a combined anterior cervical access with Median Sternotomy and anterior thoracotomy, known as the ‘trap door technique’. Among 17 patients, gross total resection was achieved in all, with negative margins in 15 cases. Importantly, no perioperative mortality or inadvertent neurovascular injuries occurred, and the sternoclavicular joints were preserved [44].

However, this technique is technically demanding, requiring advanced anatomical knowledge and surgical expertise, especially in combined cervical and thoracic procedures. Multiple incisions also increase the risk of intraoperative bleeding, wound infection, and delayed recovery, with patients experiencing greater postoperative pain and discomfort. Consequently, the combined approach is best suited for patients with large tumors, complex anatomy, or lesions involving critical structures.

## 4. Criteria for choosing different surgical approaches

In the surgical treatment of anterior mediastinal tumors, selecting an appropriate surgical approach is crucial for improving surgical outcomes, reducing complications, and accelerating recovery. The choice of approach is influenced by several factors, including the tumor’s location and size, the patient’s clinical condition, and the expected postoperative recovery.

### 4.1. Tumor location and size

Tumor’s location and size are critical factors in selecting the surgical approach. The anterior mediastinum lies adjacent to vital structures such as the heart, great vessels, and trachea, and the tumor’s relationship to these directly impacts surgical difficulty and approach. Studies suggest that tumors ≤5 cm without significant invasion of critical structures (e.g. great vessels, trachea) are considered candidates for minimally invasive approaches such as VATS or Robot-Assisted Surgery, whereas larger or invasive tumors generally require open resection to ensure both safety and completeness of the procedure [38–40].

For tumors behind the sternum or near major vessels, a Median Sternotomy remains the traditional approach, offering the widest exposure and facilitating complete resection, though with greater trauma and longer recovery. Tumors near the trachea or esophagus pose higher risks; in such cases, Thoracoscopic or Robot-Assisted Approaches enable more precise dissection through small incisions, better protecting surrounding structures while minimizing invasiveness.

### 4.2. Patient’s clinical condition

The patient’s clinical condition is central to selecting a surgical approach. Minimally invasive techniques (VATS or robot-assisted) are preferred for elderly, frail, or cardiopulmonary-compromised patients, as they reduce trauma, recovery time, and complications. Tolerance to positioning and anesthesia must also be considered, with less demanding options such as axillary or other minimally invasive access often more suitable.

In patients with myasthenia gravis (MG), three additional considerations influence the choice of approach. First, the extent of resection is usually a total thymectomy, including the thymus and surrounding perithymic fat, particularly when thymoma is present, to optimize symptom control and reduce the risk of MG recurrence. Second, the Subxiphoid Approach can be advantageous because it provides a midline view of both phrenic nerves, facilitating their preservation and thereby lowering the risk of postoperative respiratory compromise. Third, when the tumor capsule is fragile or the lesion abuts major mediastinal vessels, the selected approach must reliably allow R0 resection and secure vascular control; in such settings, open approaches such as Median Sternotomy or Cervicothoracic Incisions are often preferred, whereas minimally invasive techniques are best reserved for well-encapsulated tumors without clear evidence of invasion.

### 4.3. Postoperative recovery and complications

The surgical approach influences both recovery and complication rates. Median Sternotomy often requires prolonged hospitalization due to pain and limited mobility, whereas Thoracoscopic and Robot-Assisted Surgery, with smaller incisions and greater precision, enable faster recovery and earlier discharge. Open surgery carries higher risks of infection and bleeding, while minimally invasive techniques generally have fewer complications in patients with good pulmonary function. For complex tumors, however, limited space may increase the risk of incomplete resection or injury. Postoperative care also differs: open surgery focuse on sternal healing, while minimally invasive surgery emphasizes airway and pulmonary recovery.

**Figure 3.**
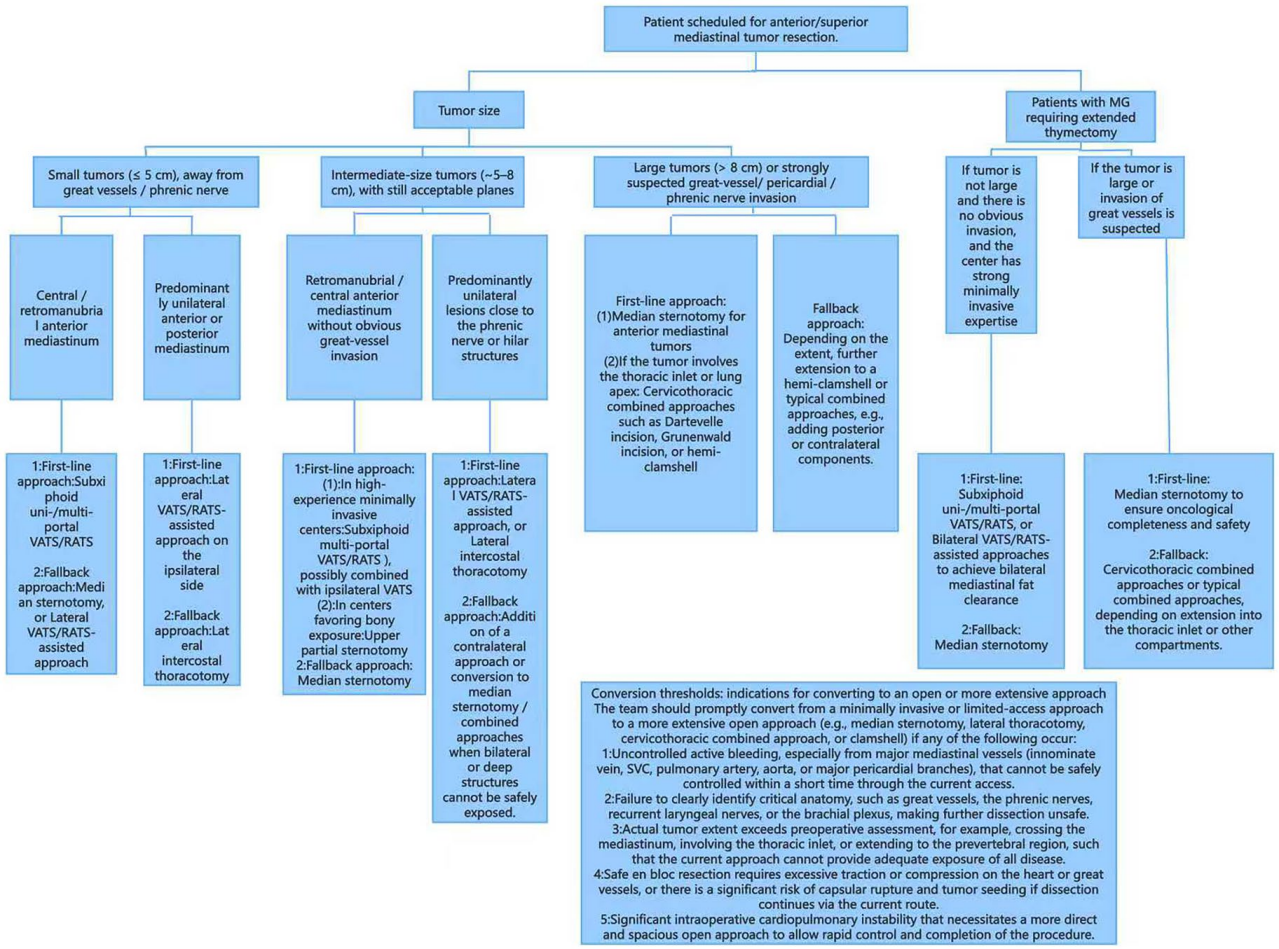
Flowchart for selecting surgical approaches in mediastinal tumor resection.

To further standardize the decision-making process for surgical approach selection, a structured flowchart (Figure 3) has been developed, which integrates the aforementioned factors (tumor size, presence of MG, tumor location, and institutional minimally invasive expertise) to guide clinicians in prioritizing first-line and fallback approaches. This flowchart also clarifies specific conversion thresholds for switching from minimally invasive to open approaches, ensuring surgical safety and oncological completeness.

Meanwhile, to provide a comprehensive comparison of the core characteristics of each surgical approach, Table 1 summarizes key parameters including exposure range, typical indications, technical points, common pitfalls/complications, conversion triggers, and supporting references. This table serves as a quick reference for clinicians to evaluate and select the most appropriate approach based on individual patient conditions and tumor features.

**Table 1.** Comparison of surgical approaches for mediastinal tumor resection.

Surgical approach	Exposure	Typical indications	Technical points	Common pitfalls/complications	Conversion triggers	Key references*
Median sternotom**y**	Wide exposure of anterior & superior mediastinum, thymus, great vessels, both phrenic nerves	Large anterior mediastinal tumors; invasive thymic tumors; complex cases needing vascular/pericardial procedures	Full midline sternotomy; wide retraction; en bloc resection; careful protection of great vessels and phrenic nerves	Greater trauma and pain; pulmonary complications; sternal infection/nonunion; risk of myasthenic crisis	Not usually needed; may add bilateral anterior thoracotomy (“clamshell”) for very extensive disease	[6,7,45]
Upper partial sternotomy: L / reverse-L / “⊥”	Good view of upper anterior mediastinum and thoracic inlet; limited caudal exposure	Medium-sized anterior/superior mediastinal tumors, retrosternal goiter, selected thymic lesions	Partial upper sternotomy in L / reverse-L or “⊥” fashion; preserve sternoclavicular joint when possible; sternum可extend to full sternotomy if needed	Limited exposure to posterior/deeper structures; risk of sternal instability, wound problems, incomplete resection	Inadequate exposure, dense adhesions, or bleeding → extend to full Median Sternotomy	[9,10]
Transverse sternotomy	Broad access to upper mediastinum and aortic arch; can be combined with bilateral anterior thoracotomies (clamshell)	Selected complex tumors near ascending aorta / arch; cases requiring combined aortic or arch-vessel reconstruction	Transverse division of upper sternum; rigid fixation at closure; may extend laterally to form hemi- or full clamshell	Larger chest wall trauma; more pain; higher risk of nonunion, chest wall deformity, pulmonary complications	Persistent poor exposure or bilateral disease → extend to hemi-/full clamshell or add intercostal incisions	[31–34]
Lateral intercostal thoracotomy	Excellent exposure of ipsilateral anterior or posterior mediastinum; bilateral thoracotomy can expose both thymic beds	Unilateral mediastinal tumors (anterior or posterior); bilateral thoracotomy for extensive thymic disease in MG	Incision usually in 3rd–4th interspace; protect intercostal neurovascular bundle; use rib spreader for exposure	Chronic post-thoracotomy pain; limited access to contralateral mediastinum; higher pain and respiratory compromise with bilateral thoracotomy	Tumor crossing midline or poor visualization → add contralateral thoracotomy or convert to sternotomy/combined approach	[34]
Lateral VATS/RATS-assisted approach	Magnified ipsilateral view of thymus, pericardium, great vessels, and phrenic nerve; bilateral VATS possible in selected cases	Small–medium anterior mediastinal tumors; early thymoma; benign cysts; selected posterior neurogenic tumors	Strategic port placement; early identification of phrenic nerve; en bloc specimen retrieval in a bag; RATS for 3D vision and wristed instruments	Limited contralateral visualization; more difficult control of major bleeding; risk of phrenic nerve injury and incomplete clearance	Uncontrolled bleeding; unclear anatomy; inability to safely dissect tumor → enlarge incision or convert to open thoracotomy / Median Sternotomy	[20,36–39,45–47]
Subxiphoid uni-/multi-portal VATS/RATS	Central, panoramic view of anterior mediastinum, thymus, pericardium, and both phrenic nerves; good retrosternal access	Early anterior mediastinal thymic lesions and prepericardial masses without great-vessel invasion	Small subxiphoid incision ± bilateral subcostal ports; create retrosternal space; elevate lower sternum; adapt to “from below” view; RATS may enhance dexterity	Possible hemodynamic instability from heart compression; narrow space in extreme body habitus; high risk if tumor invades great vessels	Inadequate cranial/lateral exposure or major bleeding from great vessels → convert to partial/full sternotomy or add intercostal approach	[5,34,42]
Kocher incision; anterior “T” cervical–sternal incision	Kocher: good access to retrosternal upper mediastinum and cervical structures above aortic arch; “T” incision extends view into upper anterior mediastinum	Retrosternal goiter and small upper mediastinal lesions for Kocher; larger cervicothoracic goiters or tumors for anterior “T”	Kocher: transverse neck incision, mobilize thyroid/retrosternal mass, protect recurrent laryngeal nerves; “T”: add limited upper sternotomy with small retractor and secure closure	Limited inferior exposure; risk of recurrent laryngeal nerve injury, hypocalcemia; “T” shares sternal wound risks; two incisions increase wound issues	Incomplete mobilization, poor visibility, or bleeding → convert to upper or full Median Sternotomy	[11–19]
Dartevelle, Grunenwald, hemi-clamshell and other cervicothoracic combined approaches	Excellent exposure of thoracic inlet, subclavian and innominate vessels, brachial plexus, lung apex, and upper mediastinum	Complex cervicothoracic and superior sulcus tumors, invasive thymic or lung tumors involving thoracic inlet vessels/plexus or spine	Often involve partial clavicle/1st-rib resection or elevation; upper sternotomy plus anterolateral thoracotomy for hemi-clamshell; require preplanned sequence of vascular control, resection, and reconstruction	Major trauma, long operations, high blood loss; risk of brachial plexus injury, major vascular complications, chest wall deformity, shoulder dysfunction, respiratory failure	If caudal or contralateral spread is greater than expected, may extend to full clamshell or add posterior approaches; uncontrolled bleeding requires prompt further enlargement	[5,23,24,26–30,48]
Typical combined approaches	Complementary views (e.g. cervical for neck/upper mediastinum + subxiphoid for anterior mediastinum; subxiphoid + intercostal for anterior + lateral mediastinum)	Multicompartment disease (neck + mediastinum; bilateral mediastinal fat in MG; tumors crossing compartments) where one route alone is insufficient	Careful preoperative planning of each incision’s role and sequence; coordinated traction from different directions; positioning must accommodate all approaches	Increased complexity; higher risk of uncoordinated traction, longer operative time, and cumulative wound-related morbidity	Persistent inadequate exposure or bleeding despite combined approaches → convert to more extensive open procedure (full sternotomy, extended thoracotomy, clamshell)	[44]

## 5. Conclusion

Surgical treatment of anterior mediastinal tumors requires careful consideration of tumor size, location, patient status, and expected outcomes. The main approaches—open surgery, thoracoscopy, and Robot-Assisted Surgery—each have strengths and limitations. Median Sternotomy offers wide exposure but is highly invasive, with longer recovery and higher complication rates. Axillary incisions cause less visible trauma but have narrow applicability. Thoracoscopic Surgery, with smaller incisions, faster recovery, and fewer complications, is becoming the preferred option, though technically demanding and limited by tumor size and location. Robot-Assisted Surgery provides precise control and better visualization, particularly in complex cases, but its high cost limits widespread use. In practice, the choice of approach is also influenced by surgeon experience and institutional resources, with more advanced techniques suited to experienced teams and well-equipped institutions.

## 6. Future outlook

With the rapid development of minimally invasive surgery and Robot-Assisted Surgery, the surgical treatment of anterior mediastinal tumors is expected to become more precise and personalized. Continuous improvement of robotic technology and intelligent assistance systems are anticipated to further enhance surgical safety and accuracy, reduce the incidence of complications, and shorten hospital stays and recovery periods. Meanwhile, the ongoing advancements in imaging technologies and intraoperative navigation systems will play increasingly important roles in preoperative planning, intraoperative procedures, and postoperative assessment. Specifically, artificial intelligence (AI) is poised to revolutionize surgical planning by enabling automated tumor segmentation, risk stratification, and personalized approach optimization based on multi-modal clinical data, while augmented reality (AR) could provide real-time anatomical overlays and intraoperative guidance, bridging the gap between preoperatively mapped structures and intraoperative visualization to improve precision and reduce procedural errors.

## 7. Limitations

This study is a narrative review, and as such, it is subject to selection bias, as it relies on available literature rather than systematic or meta-analytic methods. Additionally, heterogeneity across centers and the lack of pooled estimates limit the ability to draw definitive conclusions about the optimal surgical approach. These factors should be considered when interpreting the findings.

## Data Availability

Data sharing is not applicable to this article as no data were created or analysed in this study.
